# Sporadic inclusion body myositis: no specific cardiac involvement in cardiac magnetic resonance tomography

**DOI:** 10.1007/s00415-020-09724-4

**Published:** 2020-01-29

**Authors:** Angela Rosenbohm, Dominik Buckert, Jan Kassubek, Wolfgang Rottbauer, Albert C. Ludolph, Peter Bernhardt

**Affiliations:** 1 Department of Neurology, University of Ulm, Oberer Eselsberg 45, 89081 Ulm, Germany; 2 Department of Internal Medicine II, University of Ulm, Ulm, Germany; 3 Heart Clinic Ulm, Ulm, Germany

**Keywords:** Inclusion body myositis, Cardiac magnetic resonance tomography, Late gadolinium enhancement, Myocarditis

## Abstract

**Objective:**

To investigate cardiac involvement in patients with sporadic inclusion body myositis (IBM) by cardiac magnetic resonance tomography (CMR).

**Methods:**

A case series of 20 patients with IBM underwent basic cardiac assessment and CMR including functional imaging, native and contrast-enhanced T1-weighted, and late gadolinium enhancement (LGE) imaging.

**Results:**

All IBM patients reported no cardiac symptoms. Echocardiography was normal in 16/17 IBM patients. In CMR, IBM patients had normal ejection fractions (mean LVEF 63 ± 7%) and ventricular mass. They had reduced left (mean 55 versus 88 ml) and right ventricular stroke volumes (mean 54 versus 86 ml) and increased early myocardial enhancement (pathological T1 Ratio in 44% versus 5%), as compared to age- and gender-matched controls. Since arterial hypertension was more often observed in IBM patients, hypertensive heart disease can also be causative for these changes. Late gadolinium enhancement did not differ statistically from healthy controls. There was no apparent association between elevated biomarkers, echocardiography and CMR.

**Conclusion:**

CMR revealed subtle changes in cardiac geometry and tissue characterization in IBM patients when compared to a gender- and age-matched control group. Findings in CMR indicated a higher extent of diffuse myocardial fibrosis as well as smaller left ventricular stroke volumes. These alterations may be due to a higher prevalence of arterial hypertension in the IBM cohort.

## Introduction

Sporadic inclusion body myositis (IBM) is the most common and disabling inflammatory myopathy among persons > 50 years of age [1, 2]. Inclusion body myositis shows a highly characteristic pattern of weakness with selective involvement of long finger flexors and quadriceps muscle as well as dysphagia in some patients. The pathogenic origin of the disease is still unknown [3–5]. Anti-dysimmune treatments are generally not effective and were even reported to be detrimental for IBM patients [6], which distinguishes IBM from other primary inflammatory myopathies. Current diagnostic criteria involve clinical and pathological data in making the diagnosis [7]. Cardiac involvement was scarcely reported in single case reports concerning dilatative cardiomyopathy [8], ventricular hypertrabeculation [9], subepicardial fatty replacement [10], ventricular tachycardia [11], atrioventricular blocks [12], and hypertrophic cardiomyopathy [13].

Epidemiologic studies found no significant increase of cardiovascular disease in IBM patients since cardiac abnormalities would also be expected in healthy controls of this age group [5, 14]. In summary, cardiac involvement in IBM is not a frequent finding. However, objective diagnosis, especially in subclinical manifestation, is not ascertained yet and hard to distinguish from age-dependent cardiovascular alterations in an elder patient cohort.

Cardiac magnetic resonance (CMR) imaging has been shown to be a valuable tool in the diagnostic work-up of inflammatory myocarditis [15, 16] and for the detection of cardiac involvement in systemic muscle diseases [17–19]. CMR has been used as an objective diagnostic tool in the detection of cardiac involvement in IBM in two singular case reports [8, 10], detecting cardiomyopathy and fatty replacement of heart muscle, respectively. Early diagnosis of heart muscle involvement of myopathies can result in early therapy of heart failure, leading to beneficial ventricular remodeling, and thus less left ventricular dysfunction and consequently improvement of survival in these patients. The aim of this study was to characterize potential cardiac involvement in IBM using a comprehensive CMR protocol.

## Methods

### Patients and controls

Patients with histologically proven inclusion body myositis [20] were screened for enrollment into the study. All patients underwent skeletal muscle biopsy for histopathological diagnosis of the underlying disease. The European Neuromuscular Center (ENMC) research criteria were used to make a clinico-pathologically defined diagnosis or a clinically defined IBM.

Medical charts were reviewed for clinical and laboratory data. Exclusion criteria were contraindication for CMR, gadolinium-based contrast agent, pregnancy, or no secured contraception. All included patients gave informed written consent. The study was approved by the local ethics committee (reference no. 13/10).

Blood levels of creatine kinase (CK), myocardial creatine kinase (CK-MB), troponin I and N-terminal prohormone of brain natriuretic peptide (NT-proBNP) were analyzed for each patient (normal range: CK < 171 U/l, CK-MB < 25 U/l, NT-proBNP 0–125 pg/ml).

As controls, a cohort of age- and gender-matched patients from the ongoing multicenter study for the assessment of age- and gender-specific reference values for cardiac imaging markers (USAGE) was implemented. Within this study, healthy subjects lacking any cardiac comorbidity underwent a comprehensive CMR examination.

### Transthoracic echocardiography

Transthoracic echocardiography (2D and Doppler) was performed using a CX 50 Ultrasound (Philips Healthcare Germany). Left ventricular (LV) cavity dimensions, mass and wall thickness, and diastolic dysfunction were assessed in accordance with the recommendations of the European Association of Echocardiography [20].

An abnormal echocardiography was defined by a left ventricular end-diastolic diameter (LVEDD) > 56 mm, an interventricular septum (IVS) > 11 mm and a left ventricular end-systolic diameter > 40 mm. The ejection fraction was visually evaluated. Additionally, echocardiography was used to assess valve disease and fractional shortening.

### CMR study

All patients were examined in a 1.5 T whole-body scanner (Intera, Philips Medical Systems, Best, the Netherlands) using a 32-channel phased-array cardiac surface coil. Steady-state free precession cine sequences were acquired in contiguous short axis orientation covering the left and right ventricle for volumetric and functional analysis of both ventricles (repetition time 3.4 ms, echo time 1.7 ms, slice thickness 8 mm, no interslice gap, acquisition in end-expiration breath-holds) as previously reported [21].

A non-breath-hold T1-weighted fast spin-echo sequence was performed in three axial slices using the body coil (echo time 25 ms, flip angle 90°, repetition time 1xRR). This sequence was acquired before and 30 s after intravenous application of 0.1 mmol gadolinium-based contrast agent (Dotarem, Guerbet, Villepinte, France). After acquisition of T1-weighted images, a second bolus of 0.1 mmol contrast agent was injected [21]. Ten minutes later, an inversion-recovery gradient-echo sequence for evaluation of late gadolinium enhancement (LGE) was acquired in contiguous short-axis orientation covering the entire left ventricle (repetition time 7.1 ms, echo time 3.2 ms, slice thickness 8 mm, respiratory navigator, inversion time was individually adjusted for complete nulling of the myocardium) [21].

### CMR analysis

CMR images were anonymized and transferred as DICOM images to a dedicated workstation. Two blinded and experienced readers evaluated all images in consensus using commercially available software (cmr42, Circle, Cardiovascular Imaging, Calgary, Canada). End-diastolic and end-systolic endocardial contours of the left and right ventricle were drawn manually for evaluation of end-diastolic and end-systolic volumes. Ejection fractions were calculated, and end-diastolic left-ventricular epicardial contours were drawn for assessment of left ventricular myocardial mass. Stroke volumes and myocardial mass were also reported as indexes (relation to body surface area as a matter of body weight).

Regions of interest covering the left ventricular myocardium were drawn in the pre-contrast T1-weighted images and copied to the postcontrast images. Early myocardial enhancement was calculated in accordance to the Lake Louis Criteria as increase of signal intensity in percent [16]. For T1 ratio, values > 4 were considered pathological. These criteria are well established and suggested for CMR diagnostic work-up of inflammatory myocardial disease [16].

The inversion-recovery gradient-echo sequence was evaluated for presence of hyperenhancement consistent with myocardial fibrosis. LGE volume was quantified as percentage of left ventricular myocardial using a cutoff signal intensity increase of more than five standard deviations of remote myocardium [22].

### Statistical analysis

All data are reported as median value and interquartile range [Q1, Q3] in case of normal distributed values with mean and standard deviation. Mann–Whitney *U* test was used for non-normal distributed variables. A *p* value ≤ 0.05 was regarded to be statistically significant.

Results from all tests were considered exploratory, in keeping with the study design and therefore, no adjustment for multiple testing was done.

## Results

### Patients and diagnosis

The study group consisted of 20 patients with histologically proven IBM. 14 patients were scored as clinico-pathologically defined diagnosis and 6 patients as clinically defined IBM. Thirteen patients were treated with immunoglobulins (IVIG) every 6–8 weeks during the CMR acquisition, one of the patients was on therapy with corticosteroids and mycophenolate mofetil during the 3 months before CMR. All patients fulfilled the diagnostic ENMC criteria [7] at the time of CMR. There were no other autoimmune disorders reported from all of the IBM patients. Mean age of the study patients was 61 years, 35% were female. Patients’ characteristics including cardiovascular risk factors, blood levels of CK, CKMB and NT-pro BNP, and myopathy symptoms are provided in Table 1.

**Table 1:** Clinical characteristics of IBM patients and controls

	IBM patients (*N* = 20)	Controls (*N* = 20)	Unpaired *t* test *p* value
Age (years), mean ± SD	61.4 ± 12.3	68.06 ± 9.59	0.83
Females, *n* (%)	7 (35)	7 (35)	1.00
Arterial hypertension (AHT), *n* (%)	13 (65)	5 (25)	**0.01**
Diabetes, *n* (%)	2 (10)	1 (5)	0.08
CK (norm < 171), (U/l)	613 ± 467	–	–
CKMB (norm < 25) (U/l)	37 ± 21	–	–
Troponin I (norm < 14) (ng/l)	4.0 ± 4.0	–	–
NT-pro BNP (norm 0–125) (pg/ml)	232 ± 389	–	–
Angiotensin-converting enzyme (ACE) inhibitor or angiotensin 1 receptor blocker (*n*)	10	–	–
Beta-blocker (*n*)	8	–	–
Other antihypertensive medications (*n*)	9	–	–
AVB grade II (*n*)	0	–	–
Atrial fibrillation (*n*)	0	–	–
Supraventricular tachycardia (*n*)	0	–	–

Bold value indicate* p* < 0.05

*NSVT* non-sustained ventricular tachycardia, *AVB* atrioventricular block, *VPC/h* ventricular premature contractions/hour

### Echocardiography

Echocardiography revealed normal diameters of the ventricles in all patients. The systolic function was not diminished in the visual evaluation with normal values of fractional shortening in 16/17 patients. Slight insufficiencies of the valves were reported in several patients. No pericardial effusions were reported. All other documented values were in the normal range despite an increase of left atrium diameter in 4/17 patients (Table 2).

**Table 2:** Results of echocardiography in 17 IBM patients

	IBM patients
LA (norm < 40), mean ± SD (mm)	40.1 ± 5.2
LVEDD (norm < 56 mm), mean ± SD (mm)	50.8 ± 6.7
LVESD (norm < 42 mm), mean ± SD (mm)	31.4 ± 5.5
FS fractional shortening (LVEDD–LVESD) (norm > 25%), mean ± SD (%)	37.4 ± 10.5
IVSDD (norm 5–11 mm), mean ± SD (mm)	10.1 ± 2.0
AI (slight) (*n*)	4/17
MI (slight) (*n*)	13/17
TI (slight) (*n*)	12/17
PI (slight) (*n*)	5/17

*LA* left atrium, *LVEDD* left ventricular end-diastolic diameter, *LVESD* left ventricular end--systolic diameter, *FS* fractional shortening, *IVSDD* intraventricular septum end-diastolic diameter, *AI* aortic insufficiency, *MI* mitral insufficiency, *TI* tricuspidal insufficiency, *PI* pulmonary insufficiency

### Blood tests

Creatine kinase was elevated in 18/20 patients (mean 613 ± 467 U/L, normal range < 171 U/l) and CKMB in 11/20 (mean 37 ± 21 U/L, normal range < 25 U/l). Troponin I was normal in all patients tested (*N* = 11 at time of MRI). NT-proBNP (with age- and gender-correlated normative values) was abnormal in 3/15 patients, indicating myocardial insufficiency.

### CMR data

**Fig. 1:**
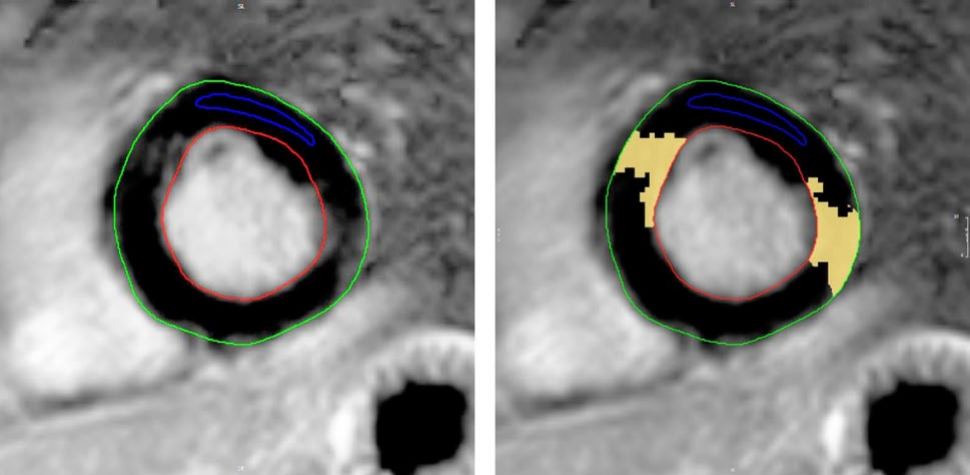
Early gadolinium enhancement (EGE) in transversal orientation. Relative myocardial enhancement 60.1%, ratio of EGE (myocardium/skeletal muscle) 4.3

**Fig. 2:**
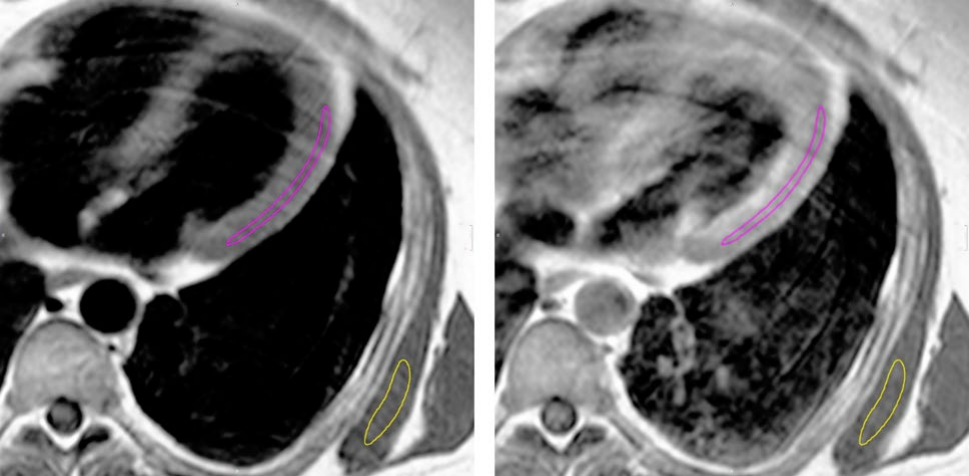
CMR images of typical observed alterations in IBM. Late gadolinium enhancement (LGE) in short-axis orientation. Red and green contours indicate endocardial and epicardial borders, respectively. There is a patchy intramural contrast enhancement in the anteroseptal and inferolateral segments (yellow area on right image)

CMR was completed in 20 IBM patients and 20 controls. Mean left and right ventricular ejection fractions (LVEF, RVEF) were in the normal range (Table 3), but myocardial stroke volumes were significantly reduced in IBM patients compared to the control group (both left and right ventricular stroke volume and indexes, respectively). The mean left ventricular ejection fraction was 63% (range 47–73). Early myocardial gadolinium enhancement in T1 could be observed in 8/18 (44%) of IBM patients and in 1/19 controls, resulting in a significant difference (Fig. 1). LGE patterns consistent with a myocardial fibrosis were detected in 7 (35%) patients, but this abnormality did not reach significance, since 4 controls also had a pathological LGE. In IBM patients, LGE was distributed in the inferior and inferolateral segments (Fig. 2). These LGE patterns are not characteristic for an ischemic origin, but are commonly reported in inflammatory myocarditis or cardiac involvement in systemic diseases [18, 23].

**Table 3:** CMR characteristics of patients and controls

	Unit	IBM cohort (*n* = 20)	Control cohort (*n* = 20)	Unpaired *t* test *p* value
LVEDV (ml)	Median [5;95]	136.5 [81.3; 305.0]	153.5 [82.4; 238.0]	0.52
LVEDV index (ml/m^2^)	Mean ± SD	73.2 ± 20.7	76.15 ± 17.25	0.63
LVSV (ml)	Median [5;95]	49.8 [24.1; 129.4]	87.0 [39.6;130.9]	**0.0001**
LVSV index (ml/m^2^)	Median [5;95]	25.5 [14.1; 4.0]	45.0 [19.6; 72.1]	** < 0.0001**
Ventricular mass (g)	Mean ± SD	107.1 ± 37.5	100.3 ± 23.4	0.51
Ventricular mass index (g/m^2^)	Median [5;95]	51.5 [35.1; 112.0]	49.5 [2.1; 77.4]	0.28
LVEF (%)	Mean ± SD	62.60 ± 6.531	59.9 ± 6.7	0.20
RVEDV (ml)	Mean ± SD	139.1 ± 47.2	150.8 ± 41.5	0.41
RVEDV index (ml/m^2^)	Mean ± SD	70.2 ± 17.3	77.3 ± 18.3	0.22
RVSV (ml)	Mean ± SD	53.5 ± 20.8	85.7 ± 23.0	** < 0.0001**
RVSV index (ml/m^2^)	Mean ± SD	26.8 ± 8.5	44.1 ± 11.2	** < 0.0001**
RVEF (%)	Mean ± SD	62,04 ± 6,637	61.1 ± 10.3	0.72
T1 ratio (normal < 4.0)	Median [5;95]	3.8 [2.0; 11.0]	2.9 [1.0; 6.4]	0.10
T1 ratio path, *n* (%)	Frequency	8/18 (44)	1/19 (5)	**0.005**
PE, *n* (%)	Frequency	3 (15)	4 (20)	0.69
LGE, *n* (%)	Frequency	7 (35)	4 (20)	0.30

*AHT* arterial hypertension, *LVEDV* left ventricular end-diastolic volume, *LVSV* left ventricular stroke volume, *LVEF* left ventricular ejection fraction, *RVEDV* right ventricular end-diastolic volume, *RVS*V right ventricular stroke volume, *PE* pericardial effusion, *LGE* late gadolinium enhancement

Significant *p* values are indicated in bold

CMR indexes are related to body surface area (BSA), which was calculated by the Dubois and Dubois regression formula BSA = 0.007184 × weight(kg)^0.425^ × height[cm]^0.725^

Patients with and without LGE did not differ significantly for all other variables.

Patients with increased early myocardial enhancement or LGE did not show any statistical correlation with any reported laboratory value, echocardiographic parameters or any of the reported CMR analyses.

Pericardial effusion without a hemodynamic restriction were detected in the same frequency as in healthy controls. The detected pericardial effusions in the CMR were not visible in the echocardiography.

## Discussion

To the best of our knowledge, the current study is the first report of an IBM patient cohort using CMR for the detection of potential myocardial involvement. No significant abnormalities in the routine cardiac assessment with echocardiography could be found except slight valve insufficiencies in a majority of the patients.

Cardiac evaluation of 20 IBM patients with CMR revealed reduced stroke volumes, while ejection fractions and left ventricular heart mass were normal. Since the heart scales with the size of the body and therefore with height and weight, we used the index of ventricular mass and volume as a ratio of body surface area (BSA) for comparison with controls (Index = mass/BSA or volume/BSA) [24]. Indexes also revealed reduced left and right ventricular stroke volumes, while the ventricular heart mass index was normal. LGE patterns consistent with local myocardial fibrosis and scar did not differ from controls, while pathological T1 ratios indicating diffuse myocardial fibrosis were significantly increased.

A confounding factor for this difference could be the treatment (most of cases with IVIG) in the IBM cohort compared to controls. The LGE prevalence did not relate to IVIG treatment.

Though there are seven case reports with different cardiac abnormalities in IBM patients in the literature, there is only one cohort study with a systematic cardiac evaluation in 51 patients [14] and an epidemiological survey on death certificates observing 64 IBM patients [5]. In the cohort of 51 patients [14] which underwent ECG and laboratory results (CKMB, troponin T (cTnT) and I and echocardiography), the prevalence of cardiac abnormalities in IBM was not higher than would be expected in this age cohort. There was no apparent association between elevated biomarkers (CK-MB and cTnT), ECG or echocardiographic abnormalities. In a 12-year follow-up observation in the Netherlands, cardiovascular causes of death in IBM patients were comparable to the frequencies found in the age-matched overall population [5]. Patients tended to die less often from cardiovascular diseases; however, statistical significance could not be substantiated.

For arterial hypertension, left ventricular hypertrophy and reduced stroke volumes due to afterload increase are well known [25–27].

LGE may be attributed to unrecognized cardiac events (myocarditis, infarction) and hypertension [28, 29]. Since the frequency of arterial hypertension differed in cases and controls (65% versus 25%), some of the reported abnormalities in IBM patients might be due to hypertensive heart disease. With respect to CMR, increase in left ventricular mass has been described as a feature of hypertensive heart disease [26] as well as a lack of midwall LGE. Myocardial LGE consistent with myocardial fibrosis has been described in many forms of heart disease, and is frequently reported in hypertensive heart disease [30]. Long-standing hypertension is characterized by the development of structural remodeling of the myocardium, including development of left ventricular hypertrophy and diffuse interstitial fibrosis [31].

In summary, we therefore conclude that the apparent differences we found in heart ventricles and the detection of myocardial fibrosis are probably due to increased amount of arterial hypertension in our IBM cohort and not due to primary heart muscle inflammation.

It remains unclear if arterial hypertension is generally more common in IBM compared to healthy controls or if this is just a bias of the cohort sample size. In a survey on idiopathic inflammatory myositis and their comorbidities in Australia, the IBM subgroup also showed arterial hypertension in 65% (*n* = 65) [32]. A Brazilian cohort (*n* = 18) reported arterial hypertension in 72% [33].

Any form of cardiac inflammation as reported in other inflammatory muscle diseases could be ruled out in IBM. This is well in accordance to the assumption that IBM differs from primary inflammatory muscle diseases.

## References

[1] Dalakas MC (1991). Polymyositis, dermatomyositis, and inclusion-body myositis. N Engl J Med.

[2] Dalakas MC (2015). Inflammatory muscle diseases. N Engl J Med.

[3] Askanas V, Engel WK, Nogalska A (2015). Sporadic inclusion-body myositis: a degenerative muscle disease associated with aging, impaired muscle protein homeostasis and abnormal mitophagy. Biochim Biophys Acta.

[4] Dalakas MC (2006). Sporadic inclusion body myositis—diagnosis, pathogenesis and therapeutic strategies. Nat Clin Pract Neurol.

[5] Cox FM, Titulaer MJ, Sont JK, Wintzen AR, Verschuuren JJ, Badrising UA (2011). A 12-year follow-up in sporadic inclusion body myositis: an end stage with major disabilities. Brain.

[6] Benveniste O, Guiguet M, Freebody J, Dubourg O, Squier W, Maisonobe T, Stojkovic T, Leite MI, Allenbach Y, Herson S, Brady S, Eymard B, Hilton-Jones D (2011). Long-term observational study of sporadic inclusion body myositis. Brain.

[7] Rose M.R. (2013). 188th ENMC International Workshop: Inclusion Body Myositis, 2–4 December 2011, Naarden, The Netherlands. Neuromuscular Disorders.

[8] Ballo P, Chiodi L, Cameli M, Malandrini A, Federico A, Mondillo S, Zuppiroli A (2012). Dilated cardiomyopathy and inclusion body myositis. Neurol Sci.

[9] Finsterer J, Stöllberger C, Höftberger R (2011). Left ventricular hypertrabeculation/noncompaction in hereditary inclusion body myopathy. Int J Cardiol.

[10] Utz W, Schmidt S, Schulz-Menger J, Luft F, Spuler S (2010). Cardiac involvement in sporadic inclusion-body myositis. Circulation.

[11] Prutkin JM, Patton KK (2009). Ventricular tachycardia in a patient with inclusion-body myositis. Pacing Clin Electrophysiol.

[12] Krendel DA, Gilchrist JM, Bossen EH (1988). Distal vacuolar myopathy with complete heart block. Arch Neurol..

[13] Inamori Y, Higuchi I, Inoue T, Sakiyama Y, Hashiguchi A, Higashi K, Shiraishi T, Okubo R, Arimura K, Mitsuyama Y, Takashima H (2012). Inclusion body myositis coexisting with hypertrophic cardiomyopathy: an autopsy study. Neuromuscul Disord..

[14] Cox FM, Delgado V, Verschuuren JJ, Ballieux BE, Bax JJ, Wintzen AR, Badrising UA (2010). The heart in sporadic inclusion body myositis: a study in 51 patients. J Neurol.

[15] Marholdt H, Goedecke C, Wagner A (2004). Cardiovascular magnetic resonance assessment of human myocarditis: a comparison to histology and molecular pathology. Circulation.

[16] Friedrich MG, Sechtem U, Schulz-Menger J (2009). International Consensus Group on cardiovascular magnetic resonance in myocarditis. Cardiovascular magnetic resonance in myocarditis: a JACC White Paper. J Am Coll Cardiol.

[17] Walcher T, Steinbach P, Spieß J (2011). Detection of long-term progression of myocardial fibrosis in Duchenne muscular dystrophy in an affected family: a cardiovascular magnetic resonance study. Eur J Radiol.

[18] Yilmaz A, Gdynia HJ, Marholdt H, Sechtem U (2009). Cardiovascular magnetic resonance reveals similar damage to heart of patients with Becker and limb-girdle muscular dystrophy but no cardiac symptoms. J Magn Reson Imaging..

[19] Rosenbohm A, Buckert D, Gerischer N, Walcher T, Kassubek J, Rottbauer W, Ludolph AC, Bernhardt P (2015). Early diagnosis of cardiac involvement in idiopathic inflammatory myopathy by cardiac magnetic resonance tomography. J Neurol..

[20] Dubowitz V, Sewry CA (2013). Histological and histochemical stains and reactions. Muscle biopsy. A practical approach.

[21] Buckert D, Dewes P, Walcher T, Rottbauer W, Bernhardt P (2013). Intermediate term prognostic value of reversible perfusion deficit diagnosed by adenosine perfusion cardiac magnetic resonance imaging—a prospective follow-up study in a consecutive patient population. J Am Coll Cardiol Imaging.

[22] Bondarenko O, Beek AM, Hofman MB (2005). Standardizing the definition of hyperenhancement in the quantitative assessment of infarct size and myocardial viability using delayed contrast-enhanced CMR. J Cardiovasc Magn Reson.

[23] Marholdt H, Goedecke C, Wagner A, Meinhardt G, Athanasiadis A, Vogelsberg H (2004). Cardiovascular magnetic resonance assessment of human myocarditis: a comparison to histology and molecular pathology. Circulation.

[24] Brumback LC, Kronmal R, Heckbert SR, Ni H, Hundley WG, Lima JA, Bluemke DA (2010). Body size adjustments for left ventricular mass by cardiovascular magnetic resonance and their impact on left ventricular hypertrophy classification. Int J Cardiovasc Imaging.

[25] Ganau A, Devereux RB, Roman MJ, de Simone G, Pickering TG, Saba PS, Vargiu P, Simongini I, Laragh JH (1992). Patterns of left ventricular hypertrophy and geometric remodeling in essential hypertension. J Am Coll Cardiol.

[26] Hoey ET, Pakala V, Teoh JK, Simpson H (2014). The role of imaging in hypertensive heart disease. Int J Angiol.

[27] Mavrogeni S, Katsi V, Vartela V, Noutsias M, Markousis-Mavrogenis G, Kolovou G, Manolis A (2017). The emerging role of cardiovascular magnetic resonance in the evaluation of hypertensive heart disease. BMC Cardiovasc Disord.

[28] Themudo R, Johansson L, Ebeling-Barbier C, Lind L, Ahlström H, Bjerner T (2017). The number of unrecognized myocardial infarction scars detected at DE-MRI increases during a 5-year follow-up. Eur Radiol.

[29] Andersen K, Hennersdorf M, Cohnen M, Blondin D, Modder U, Poll LW (2009). Myocardial delayed contrast enhancement in patients with arterial hypertension: initial results of cardiac MRI. Eur J Radiol.

[30] Díez J, González A, López B, Querejeta R (2005). Mechanisms of disease: pathologic structural remodeling is more than adaptive hypertrophy in hypertensive heart disease. Nat Clin Pract Cardiovasc Med.

[31] Schumann CL, Jaeger NR, Kramer CM (2019). Recent advances in imaging of hypertensive heart disease. Curr Hypertens Rep.

[32] Limaye VS, Lester S, Blumbergs P, Roberts-Thomson PJ (2010). Idiopathic inflammatory myositis is associated with a high incidence of hypertension and diabetes mellitus. Int J Rheum Dis.

[33] de Camargo LV, de Carvalho MS, Shinjo SK, de Oliveira ASB, Zanoteli E (2018). Clinical, histological, and immunohistochemical findings in inclusion body myositis. Biomed Res Int.

[34] Lang RM, Badano LP, Mor-Avi V, Afilalo J, Armstrong A, Ernande L (2015). Recommendations for cardiac chamber quantification by echocardiography in adults: an update from the American Society of Echocardiography and the European Association of Cardiovascular Imaging. Eur Heart J Cardiovasc Imaging.

